# A qualitative study on the current status and needs of unintentional injury prevention and control interventions for children aged 3–12 in Guizhou Province, China

**DOI:** 10.3389/fpubh.2025.1712091

**Published:** 2025-12-08

**Authors:** Xiujuan Li, Roumei Lin, Pan Wen, Liping Li, Linlin Xie, Yaogui Lu, Guanhua Fan, Zicheng Cao, Fancun Meng, Yanhong Huang, Jian-E Peng

**Affiliations:** 1 School of Public Health, Shantou University, Shantou, China; 2 Injury Prevention Research Center, Shantou University Medical College, Shantou, China; 3 Mental Health Center, Shantou University, Shantou, China; 4 College of Liberal Arts, Shantou University, Shantou, China

**Keywords:** children, unintentional injuries, prevention and control status, prevention and control needs, qualitative study

## Abstract

**Background:**

Unintentional injuries are a leading cause of death and disability among children and adolescents worldwide, particularly in economically underdeveloped regions. Guizhou, an impoverished and multi-ethnic region in southwestern China, faces elevated risks due to limited healthcare resources and a high proportion of left-behind children. However, few studies have explored prevention and control needs from the perspective of target populations. Using a qualitative approach, this study adopted a “bottom-up” perspective to investigate the needs of children, caregivers, teachers, and community workers in Guizhou regarding the prevention and control of unintentional injuries among children, aiming to provide a scientific basis for developing precise, culturally adapted intervention strategies.

**Objective:**

This study aimed to examine the current status and needs of unintentional injury prevention and control interventions for children aged 3–12 in Guizhou Province.

**Methods:**

Participants were selected using purposive sampling. From July 8 to 22, 2024, semi-structured interviews were conducted with children aged 3–12, their caregivers, teachers, and local social workers in Guiyang, Liping County, and Xingyi City, Guizhou Province. These included 31 focus group discussions with children and 121 in-depth interviews with caregivers, teachers, and community workers. Thematic analysis of the data was performed using NVivo 15 software.

**Results:**

Data analysis revealed four core themes: existing prevention and control interventions, intervention effectiveness, intervention gaps, and intervention needs. Existing interventions primarily included safety education, joint prevention and control mechanisms, and physical protection measures. While these efforts were reported to improve children’s safety awareness and reduced injury incidents, gaps remained, such as inadequate parental supervision, prominent environmental hazards, insufficient publicity, resource shortages, and poor cross-sector collaboration. Vulnerable groups, including left-behind children living alone and children with disabilities, faced higher risks. Participants expressed diverse intervention needs, including environmental modifications, resource support, enhanced publicity, and innovative prevention strategies.

**Conclusion:**

The current government-led prevention and control system has achieved certain successes but requires a shift from broad coverage to targeted interventions. Future efforts should strengthen family responsibility, improve multi-sectoral collaboration, and increase resource allocation. Integrating technology and cultural guidance to establish a multi-sectoral prevention network could reduce unintentional injury risk among children and promote health equity.

## 1 Introduction

Injuries are one of the leading causes of death and disability among children and adolescents worldwide (1). Nearly 2,300 children and adolescents die from injuries each day globally (2), with unintentional injuries accounting for over 90% of these cases (3). In China, unintentional injuries are the primary cause of death among children and adolescents (4). Unintentional injuries in children refer to harm caused to their health and lives by external, sudden, and unintended events (5). According to the International Classification of Diseases (ICD-10), unintentional injuries are categorized into 10 types based on external causes, including falls, road traffic crashes, and drowning (6). Such injuries severely affect children’s physical and mental health and may even lead to death, placing a heavy burden on families and society (7).

The occurrence of unintentional injuries in children results from multiple interacting factors, including family environment, socioeconomic status, cultural practices, and medical conditions. Mortality rates vary significantly across regions and countries (3, 7), with children in low- and middle-income countries being particularly vulnerable (6, 8). As one of the relatively impoverished provinces in southwestern China, Guizhou faces challenges such as high poverty rates and limited medical resources (9). Its multi-ethnic composition (10) further complicates the prevention and control of unintentional injuries among children. In addition, Guizhou is a major source of migrant labor, with left-behind children (defined as those with one or both parents working away from home for ≥6 months) accounting for nearly half of the rural child population (11). Due to inadequate supervision, healthcare, and educational resources, these children face elevated risks of unintentional injuries. However, there is a lack of systematic epidemiological data on injuries among children aged 3–12 years in Guizhou Province. A limited number of studies suggest that non-fatal injuries commonly include falls, animal bites, burns, and road traffic injuries (9, 12, 13). Regarding fatal injuries, research is scarcer. While research indicates that accidental suffocation, drowning, and traffic crashes constitute the leading causes of unintentional injury mortality among children under five in Guizhou (14), there remains a notable lack of specific research focusing on fatal unintentional injuries in the 3–12 age group in Guizhou province. Consequently, trends for both fatal and non-fatal injuries remain unclear, underscoring the critical need for more systematic surveillance and context-specific research.

Current domestic research on the prevention and control of unintentional injuries in children primarily focuses on evaluating the effectiveness of existing interventions (15, 16). Such top-down approaches often overlook the actual needs of target populations, such as local families, communities, and children themselves. Notably, Zhang et al. (17) used mixed methods to examine the preferences of caregivers of children aged 0–6 regarding mobile health interventions, offering a new perspective on understanding user needs. However, there remains a lack of systematic research exploring local prevention and control needs in regions like Guizhou, which are characterized by ethnic diversity and a large rural population.

Therefore, grounded in the unique sociocultural context of Guizhou, this study adopts a constructivist paradigm and qualitative research methods to address the following core questions: What is the current status and effectiveness of unintentional injury prevention and control measures for children? What gaps exist? What types of support do local children, caregivers, and community workers truly need? By uncovering these under-recognized needs, this study aims to provide a scientific basis for developing targeted, culturally adapted, and precise prevention strategies. Ultimately, it seeks to reduce the risk of unintentional injuries in children and promote health equity.

## 2 Methods

### 2.1 Study design

This qualitative study was conducted from July 8 to 22, 2024, using semi-structured interviews to collect participants’ experiences and perspectives regarding unintentional injuries among children aged 3–12. This age range was selected because it encompasses critical and distinct developmental stages: preschool (3–5 years), middle childhood (6–9 years), and pre-adolescence (10–12 years). Across this period, children experience rapid changes in mobility, independence, cognitive development, and risk perception, making it a crucial window for unintentional injury prevention research and intervention. The interviews included focus group discussions (with 169 children aged 3–12, in groups of 5–6) and in-depth interviews (with 121 adults, including caregivers, teachers, and local social workers). All interviews were conducted face-to-face in Mandarin, audio-recorded, and lasted between 30 and 45 min. The study strictly adhered to the ethical principles of the Declaration of Helsinki. All participants provided written informed consent prior to the interviews. The interviews were guided by a structured protocol developed specifically for this study (see Appendix 1), and the results are reported in accordance with the Consolidated Criteria for Reporting Qualitative Research (COREQ) guidelines (18). The study was approved by the Ethics Committee of Shantou University (Approval No.: STU2024006001).

### 2.2 Recruitment and participants

Purposive sampling was used to recruit children aged 3–12, their caregivers, teachers, school administrators, and local social workers from central Guizhou (Guiyang city), southeast Guizhou (Liping County), and southwest Guizhou (Xingyi city). The three study sites were purposively selected to capture the broad socioeconomic spectrum within Guizhou Province. Guiyang, the provincial capital, represents a highly urbanized and economically developed context, evidenced by its leading GDP per capita (¥88,846) and urbanization rate (80.52%) in 2024 (19). In contrast, Liping County exemplifies a rural, less developed, and ethnic-minority-concentrated context, with the lowest GDP per capita (¥29,077) and urbanization rate (42.17%) among the sites (20). Xingyi City was chosen to represent a rapidly urbanizing and transitioning context, with intermediate economic indicators (GDP per capita ¥55,961; urbanization rate 59.4%) positioning it between Guiyang and Liping County (21). This selection strategy allows for a comparative understanding across urban, rural, and high-migration settings. The research team collaborated closely with local social service organizations, and participants were invited to participate in voluntary interviews via WeChat and telephone. Focus group discussions were conducted with children, while in-depth interviews were used for adults. All interviews took place at social service agencies. The principle of informed consent was strictly followed; child participants’ informed consent was provided by their guardian or proxies. All participants were informed that participation in the study was voluntary and that their information would remain confidential. Inclusion criteria were (1) children aged 3–12 and their caregivers and (2) those who provided signed informed consent. Exclusion criteria were: (1) children and caregivers outside the 3–12 age range; (2) guardians who declined participation or did not provide informed consent.

### 2.3 Data analysis

Thematic analysis was conducted using the constant comparative method (22). All interviews were transcribed verbatim. Data collection and analysis occurred concurrently to iteratively inform subsequent interviews. The coding process consisted of two phases: In the first phase, two researchers (Li and Wen) independently performed open coding on five transcripts using NVivo 15. In the second phase, three researchers (Li, Wen, and Lin) collaboratively coded an additional transcript to ensure coding consistency. Team meetings were held to discuss coding outcomes and memos and refine the analytical framework (including removing themes with insufficient evidence and merging similar themes), after which Li completed the coding of the remaining transcripts. Following two thematic review meetings, two independent researchers (Meng and Cao, who had not participated in earlier stages) were invited to validate the final analytical framework. Four researchers (Li, Lin, Meng, and Cao) collectively identified core themes through discussion. Data saturation was confirmed using the principle of thematic saturation (23). All data were anonymized, with each participant assigned a unique identifier.

## 3 Results

### 3.1 Sample characteristics and distribution

The study was conducted in central (Guiyang), southeastern (Liping County), and southwestern (Xingyi City) regions of Guizhou Province. A total of 152 valid interviews were completed, including 31 focus group discussions and 121 in-depth interviews, with 290 participants enrolled. The focus group discussions involved 169 children (79 boys, 76 girls, and 14 children with disabilities), with 5–6 participants per group. The in-depth interviews included 121 adult participants, comprising 77 caregivers, 22 teachers, 7 school administrators, and 15 social workers (see Table 1).

**Table 1:** Distribution of valid samples for different interview types and different participant categories by age group.

**Age**	**Focus group interviews**					**In-depth interviews**											
	**Left-behind children**		**Non-left-behind children**		**Disabled children**	**Caregivers of left-behind children**			**Caregivers of non-left-behind children**			**Caregivers of disabled children**			**Teachers**	**School administrators**	**Social workers**
	**Girl**	**Boy**	**Girl**	**Boy**		**Father**	**Mother**	**Grand-parents**	**Father**	**Mother**	**Grand-parents**	**Father**	**Mother**	**Grand-parents**			
3–5 years old	18	20	7	8	6	3	4	4	2	5	0	1	2	0	22	7	15
3–5 years old	21	30	5	1	8	1	8	9	0	4	0	1	6	0			
3–5 years old	23	16	2	4	0	2	3	7	1	5	0	3	5	1			
Total	62	66	14	13	14	6	15	20	3	14	0	5	13	1	22	7	15

**Figure 1:**
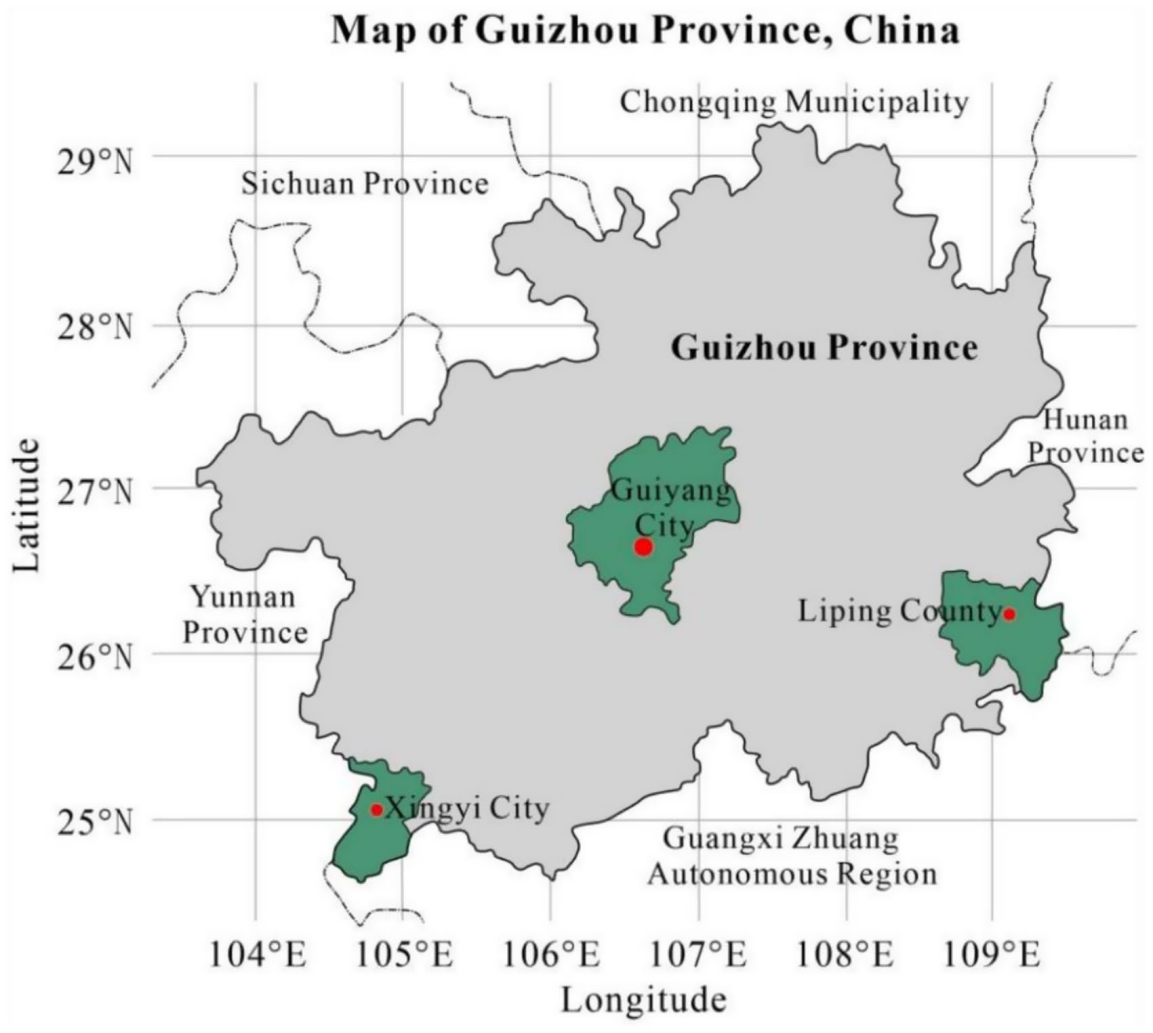
Geographic location of Guiyang City, Liping County and Xingyi City, Guizhou Province, China.

The sample covered three key demographic groups: left-behind children (primarily in Liping County and Xingyi City), non-left-behind children (mainly in Guiyang), and children with disabilities (mostly with cerebral palsy or hearing impairment). The geographical distribution of the sample is presented in Figure 1. During data collection, two interviews (involving four participants) were excluded due to poor audio quality, ensuring the reliability of the final dataset.

Through thematic analysis, we identified four core themes: (1) scope of existing prevention and control interventions; (2) reported effectiveness of interventions; (3) gaps in interventions; and (4) needs for interventions. The frequency distribution of each theme and its sub-themes is presented in Table 2.

**Table 2:** Themes and sub-themes identified.

Themes	Sub-themes (example codes)	Code frequency
Scope of existing prevention and control interventions	Safety education (daily verbal reminders; conference and lectures; social media; home visits; simulation exercises; warning signs; game-based activities; peer education; action deterrent)	739
	Joint prevention and control (home-school-community collaboration; joint patrols)	102
	Physical protection (installation of protective devices)	72
	Keep a tight watch on (−)	40
Reported effectiveness of interventions	Improved safety awareness (−)	81
	Reduction in unintentional injury events (−)	42
Gaps in interventions	Weak family guardianship (insufficient parental safety initiative; absence of guardianship; parent–child emotional alienation and blocked communication channels)	407
	Environment and facilities hidden danger (home environment and facilities hidden danger, school environment and facilities hidden danger, community environment and facilities hidden danger)	154
	Insufficient safety promotion (incomplete content; limited forms of publicity; narrow coverage)	106
	lack of professional expertise (fragmented knowledge; unsystematic training)	48
	Insufficient resources (inadequate funding; lack of human and material resources; scarce learning resources)	45
	Poor interdepartmental coordination (−)	42
	Imbalance of responsibilities and authority (−)	30
	Lack of social awareness and support (−)	29
Needs for interventions	Timely elimination of hazardous environments (−)	79
	Increased resource support (−)	77
	Enhanced publicity efforts (−)	54
	Strengthened collaboration among all parties (−)	53
	IMPROVED public facilities (−)	15
	Innovation and upgrade of prevention methods (−)	12

It is worth noting that while the themes “Gaps in Interventions” and “Needs for Interventions” are closely related, they were found to be conceptually distinct during our analysis. “Gaps” represent participants’ diagnosis of the obstacles, shortcomings, and failures within the current prevention and control system. This represents the participants’ assessment of “what is wrong with the current situation.” In contrast, “needs” represent the solutions or resource requests explicitly proposed by participants, which are future-oriented and actionable. This represents the participants’ recommendations for “what is needed in the future.”

## 3.2 Scope of existing prevention and control interventions

### 3.2.1 Safety education

Among the existing prevention and control measures, safety education is the most widespread and diverse intervention approach. At the school level, some institutions incorporate safety education into their regular curriculum, emphasizing case-based teaching and interactive experiences. At the community level, social workers employ highly interactive promotional methods such as experiential learning and peer education to make the dissemination of safety knowledge more vivid and effective. Targeting the family as a key setting, educators conduct home visits to enhance caregivers’ ability to identify and respond to risks.

“Our school holds safety education classes every Tuesday, using case videos and scenario simulations to help students visually understand the serious consequences of potential hazards.” [#130 School Administrator]

“In traffic safety education, we use experiential teaching methods, such as conducting lessons with real vehicles like buses and cars. Through experiments like ‘egg + helmet’, we demonstrate the potential injuries from falls at different heights, helping children intuitively understand the importance of safety protection and educating them not to throw objects from heights or climb dangerous elevations.” [#84 Social Worker]

“Our community implements peer education, where older children teach safety knowledge to younger ones. Due to their similar ages, older children, as educators, build trust and rapport more easily with the younger children, thereby improving the efficiency and acceptance of information delivery.”[#79 Social Worker]

“We conduct regular home visits, inspect the home environment, and immediately alert parents to any hazards we find, supervising them until corrections are made.” [#91 Social Worker]

Furthermore, family education continues to employ composite intervention strategies that combine behavior correction with positive guidance, such as implementing moderate physical punishment for dangerous behaviors to reinforce memory [Group 1, Mother of 7.18–1]. In terms of communication channels, a promotional model that integrates modern information technology with traditional environmental cues has been established. Digital platforms enable broad coverage and timely updates of safety information, while physical warning signs in the environment reinforce behavioral norms through continuous contextual cues.

*“*We update publicity boards daily with posters on preventing unintentional childhood injuries, while simultaneously sharing these safety reminders in the residents’ WeChat group to continuously reinforce the importance of safety awareness among parents and children.” [#80 Social Worker]

### 3.2.2 Joint prevention and control

The current system for preventing and controlling unintentional injuries among children in Guizhou Province operates through a multi-party collaboration model led by the government. Supported by government initiatives, schools, communities, social service organizations, and families work in coordination to establish a relatively comprehensive child safety protection network.

“The child unintentional injury prevention programs carried out by our social workers primarily rely on government support and are implemented through long-term cooperation with education departments and schools.”[#79 Social Worker]

“When organizing activities, the school collaborates with the village party secretary to assist in parental safety education, as he holds high prestige and is widely trusted within the community.” [#143 School Administrator]

“During winter and summer vacations, schools and communities jointly carry out activities such as fire safety and drowning prevention, and promote instructional videos through residents’ WeChat groups for parents to study.” [#10 Mother of non-left-behind child]

“Teachers require us parents to actively cooperate with the school’s injury prevention efforts and work together to ensure children’s safety education and supervision.” [#1 Father of left-behind child]

In terms of hazardous area management, a regular patrol system has been established. Social workers collaborate with street offices to conduct 24-h patrols in key areas such as water bodies and major traffic routes, and a rapid response mechanism has been developed to address emergencies. Additionally, to enhance parental cooperation, schools invite community authority figures such as village party secretaries to participate in safety education, leveraging their credibility and influence within the community.

“To address drowning and traffic risks, the community has established a 24-h patrol system, organizing staff to conduct regular inspections of key areas.” [#78 Social Worker]

“Our community has built a protection system involving social workers, street offices, and schools, working together to implement round-the-clock patrols and regular home visits. Furthermore, we have established a rapid response channel for emergencies to ensure timely coordination among neighborhood committees, parents, and community doctors.” [#78 Social Worker]

For key groups such as left-behind children living alone (defined as those with both parents working away from home for ≥6 months and without other guardians), schools have developed a dynamic registry shared with the community. Joint implementation of measures such as the “full-time supervision model,” “accountability system,” and “grid-based safety management system” has effectively filled gaps in family supervision, providing concrete protection for this vulnerable group.

“Our school implements a full-time supervision model for left-behind children living alone: teachers provide oversight during school hours, and social workers take over after school. Simultaneously, a grid-based management system is applied, where every 10 students form a safety group with a designated leader responsible for promptly reporting potential hazards within the group.” [#130 School Administrator]

“Our school assigns a dedicated responsible teacher to each left-behind child living alone, who conducts regular home visits and hazard inspections as part of one-on-one guardianship.” [#123 School Administrator]

### 3.2.3 Physical protection

In terms of physical protection, schools and social service agencies have widely implemented basic safety modifications. By installing protective facilities such as corner guards and safety nets, environmental hazards have been effectively eliminated.

“Our agency has installed corner guards on all table edges and protective nets on key areas such as sockets, electric fans, and windows. During home visits, we also focus on inspecting and guiding parents to eliminate potential safety hazards in the household.” [#82 Social Worker]

“Our kindergarten has laid anti-slip flooring, installed corner guards on all table edges, and placed protective locks on the hot water area of water dispensers. These measures effectively ensure children’s safety during their time at the kindergarten.” [#139 School Administrator]

“My balcony has been enclosed with guardrails by my mother, and the sharp corners of tables have been covered.” [#97 Left-behind boy]

“All the table corners in our home are covered, and protective nets have been installed on the balcony and windows.” [#10 Mother of left-behind child]

### 3.2.4 Enhanced supervision

Enhanced supervision, as a key strategy in child injury prevention, demonstrated dual characteristics of “physical supervision + technological assistance” in this study. The research found that supervisors primarily employed three methods to enforce strict oversight: first, close monitoring in daily life to ensure children remain within sight; second, the use of electronic devices such as GPS-enabled smartwatches and home surveillance cameras to assist supervision; and third, remote collaborative supervision specifically for left-behind children living alone, where parents confirm their children’s whereabouts in real-time through home monitoring devices and phone calls, repeatedly urging them to avoid dangerous areas.

“During naptime, we closely supervise the children: we prohibit them from jumping on beds to prevent falls, and also check their mouths to prevent foreign object choking.” [#147 Teacher of children with hearing impairment]

“I equipped my child with a GPS-enabled smartwatch and always check his location in real-time when he goes out.” [#22 Mother of non-left-behind child]

“Although I work in another city, I installed surveillance cameras at home. Whenever I notice through the monitor that my child is not at home, I immediately call to confirm his whereabouts.” [#3 Mother of left-behind child]

## 3.3 Reported effectiveness of interventions

Through the implementation of diversified prevention and control measures, most respondents reported a significant improvement in safety awareness among children and their guardians, which has translated into concrete changes in safety behaviors. The number of unintentional injury incidents among children in the community has decreased, and the severity of injuries has been reduced.

“The school’s daily ‘5-min safety education’ sessions have effectively enhanced students’ safety awareness and self-protection abilities.” [#139 School Administrator]

“After prolonged safety education, some students have developed a sense of safety supervision. When parents violate traffic rules, such as crossing the road during a red light, students actively dissuade them, emphasizing that ‘the teacher said we cannot go’.” [#148 School Administrator]

“Through safety education, parents now store hazardous items, such as medications and cleaning agents, properly to prevent children from accessing them.” [#92 Social Worker]

“The ‘peer education’ method, where older children teach younger ones, has been particularly effective in enhancing children’s safety awareness and improving safety behaviors.” [#79 Social Worker]

“Since our social service agency partnered with the street office to conduct 24-h patrols of dangerous roads and water bodies, there have been no cases of child drowning in our community.” [#78 Social Worker]

“After installing corner guards and shock-absorbing floor mats, the risk of injury from falls has been effectively reduced, along with the severity of injuries.” [#139 School Administrator]

## 3.4 Gaps in interventions

### 3.4.1 Inadequate family supervision

The issue of inadequate family supervision exhibits multi-dimensional and complex characteristics. Firstly, there is a widespread lack of parental safety initiative, where weak safety awareness and insufficient safety education contribute to persistent household hazards. Secondly, absentee supervision is severe: economic pressures force parents to migrate for work, resulting in a lack of direct supervision, while grandparenting often leads to lax oversight due to advanced age and heavy labor burdens among older adult caregivers. Families with children with disabilities face additional challenges due to limited care capacity.

“Many households keep knives and medicines within easy reach, and store open deep buckets of water in bathrooms.” [#85 Social Worker]

“I was stung by a bee, and my hand swelled badly. My grandmother applied tomato to my hand. When I was scalded by boiling water, she spread tea oil on it—I still have scars.” [#111 Left-behind boy]

“Left-behind children living alone in our school often engage in dangerous behaviors after school without parental supervision, such as tampering with 220-volt home circuits and even 1,000-volt high-voltage power lines, which is extremely hazardous.” [#129 School Administrator]

“Grandparent caregivers, constrained by cognitive and physical limitations, often adopt a laissez-faire approach, considering bumps and bruises a normal part of growing up.” [#92 Social Worker]

Furthermore, long-term separation between left-behind children and their parents can lead to emotional distance. This estrangement often causes children to conceal injuries rather than seek help from their parents, due to fear or reluctance. Such behavior not only delays timely treatment but also cuts off opportunities for family safety education. These three dimensions, lack of initiative, absentee supervision, and emotional distance, intertwine and reinforce each other, creating a vicious cycle of inadequate family supervision.

“After getting injured, my child did not dare to tell me, he said he was afraid I would hit him.” [#63 Mother of non-left-behind child]

“I once pinched my hand in a door, but I would not tell my parents. They work far away and are very busy, I did not want to bother them.” [#114 Left-behind girl]

“I was once scolded by my parents after my arm was crushed by a bicycle. Since then, I have not dared to tell them when I get hurt.” [#119 Non-left-behind boy]

### 3.4.2 Environmental and facility hazards

Household facility hazards are most notably represented by unsafe staircases, including wooden stairs without handrails and steep concrete stairs without protective railings, which directly increase the risk of unintentional injuries among children. It is worth noting that factors such as the simple staircases in rural wooden-structured houses and the lack of childproofing modifications in rental homes reflect the impact of economic conditions on household safety.

“The simple staircases in rural wooden houses lack handrails and proper steps, making it easy for children to miss a step and fall while going up or down.” [#145 Social Worker]

“My child once fell from an unrailed concrete staircase, but we still cannot afford to install railings.” [#71 Mother of non-left-behind child]

Community facility hazards are mainly reflected in insufficient basic protective facilities, lack of firefighting equipment, malfunctioning traffic signals, and poorly designed underground passages.

“There is a very deep well near our home without any fencing around it. It would be very dangerous if a child fell in.” [#116 Left-behind girl]

“I’ve noticed that in some older or remote communities, there are basically no firefighting facilities. If a fire broke out, the consequences would be terrible.” [#133 Teacher from a disability institution]

“Our community was relocated through poverty alleviation programs. The traffic light at the intersection is broken, making traffic crashes very likely.” [#84 Social Worker]

“The road in front of our school is on a slope. There are no traffic lights or speed bumps, making it prone to accidents.” [#129 School Administrator]

“The staircases in the underground passages in urban Guiyang have high inclination angles and are very steep, making it inconvenient for the older adults and children to walk. A fall could have serious consequences.” [#74 Father of non-left-behind child]

### 3.4.3 Insufficient safety promotion

There are significant deficiencies in child safety promotion within communities, mainly reflected in incomplete content coverage, limited forms of dissemination, and insufficient resources and capacity. Promotion efforts predominantly focus on common injury types such as drowning,traffic crashes, and falls, while paying inadequate attention to other unintentional injuries like dog bites and bee stings, as well as fire safety knowledge. Promotion targeting special groups, such as children with disabilities, is nearly absent.

“Our community mainly promotes prevention of three types of unintentional injuries: drowning, traffic crashes, and falls. Others are largely overlooked. However, incidents like dog bites and bee stings also frequently occur. Unfortunately, we are short-handed, resulting in incomplete content and limited outreach.” [#145 Social Worker]

“I believe fire safety knowledge should be promoted more, as I genuinely do not know how to use a fire extinguisher.” [#23 Mother of non-left-behind child]

“There is a lack of safety promotion for children with disabilities. We urgently need such knowledge but can only turn to the internet to learn from others’ shared experiences.” [#55 Father of a child with hearing impairment]

“Our school mainly focuses on traffic, fall, and drowning safety, with little coverage of other injuries. The promotional materials we use are downloaded from the internet, they are relatively superficial and not targeted.” [#139 School Administrator]

“The government should strengthen publicity on the prevention and control of unintentional injuries among children. The government’s high credibility would lead to better outreach outcomes. Relying solely on social workers for promotion is far from sufficient.” [#81 Social Worker from a disability institution]

### 3.4.4 Insufficient professional competence and resources

The prevention and control of unintentional injuries among children in Guizhou Province face a dual challenge. On the one hand, frontline social workers lack professional training. Some social workers have limited opportunities for training, and existing training often adopts a “secondary dissemination” model, featuring outdated content and limited coverage, which fails to meet practical needs. On the other hand, severe shortages in human and financial resources hinder the sustained and effective implementation of key measures such as in-home safety promotion, underscoring an urgent need for increased comprehensive and structural government support.

“Some social workers in our community have limited knowledge about preventing unintentional injuries among children, and learning opportunities are scarce and unsystematic.”[#91 Social Worker]

“Teachers in our institution lack professional expertise in injury prevention and control for children with disabilities. Only a few have received systematic training, while most acquire knowledge through secondary learning, resulting in inadequate mastery and affecting the effectiveness of communication with parents.” [#87 Social Worker from a disability institution]

“Publicity efforts are undoubtedly insufficient. For example, due to shortages in human and financial resources, our community agency conducts household promotion very infrequently.” [#88 Social Worker from a disability institution]

“Our agency’s promotion of unintentional injury prevention is limited due to insufficient staff and small institutional capacity.” [#145 Social Worker]

### 3.4.5 Poor interdepartmental coordination

There is currently a lack of effective collaboration among families, communities, schools, and government departments, with blurred boundaries of responsibility among parties leading to gaps in prevention and control efforts. For instance, schools reported that off-campus dangerous water bodies lacked protective measures due to unclear jurisdictional authority, resulting in drowning incidents [#129 School Administrator]. Additionally, due to limited resources, social service agencies can only prioritize serving some high-risk groups. As one school administrator noted: “Social service agencies have limited capacity and insufficient staff, they can only temporarily help us supervise left-behind children living alone, and their collaboration with us is limited.” [#143 School Administrator]. Furthermore, “when social service organizations hold promotional events, low public participation due to limited influence highlights the urgent need for the government to lead the establishment of a cross-sector collaboration mechanism that integrates resources from all parties to advance child safety.” [#85 Social Worker].

### 3.4.6 Imbalance of responsibilities and authority

Imbalance of responsibilities and authority refers to the unreasonable distribution and inadequate fulfillment of duties among families, schools, and the government in preventing and controlling unintentional injuries among children. For example, “education authorities have over-concentrated guardianship responsibilities, which should be shared by multiple parties, on schools through administrative measures such as shortening vacations” [#145 Social Service Manager] forcing schools to assume excessive supervisory duties and weakening parental guardianship. Government departments often engage in superficial promotion, as illustrated by: “When conducting safety promotion at the grassroots level, it is mostly perfunctory, taking a few photos for documentation and leaving, lacking effective supervision and failing to implement policies thoroughly.” [#81 Social Worker].

### 3.4.7 Lack of social awareness and support

Current social attention to child safety remains insufficient, manifesting a passive pattern of “neglect before incidents, emphasis after incidents,” which hinders the establishment of a long-term protection mechanism. In particular, the safety of special groups of children has not received due attention. The public lacks awareness of assistive devices for children with disabilities (e.g., hearing aids), making it difficult to foster a socially supportive environment. Furthermore, “accessible facilities are often nominal, with common issues such as occupied braille paths and overpasses lacking accessible ramps”[#132 Teacher from a disability institution], increasing daily safety risks for children with disabilities.

“We usually pay insufficient attention to unintentional injuries, they only draw concern after an incident occurs, but are soon forgotten again.” [#80 Social Worker]

## 3.5 Needs for interventions

### 3.5.1 Timely elimination of hazardous environments

Some participants reported numerous safety hazards in schools and communities, such as slippery playgrounds, water accumulation in cafeterias, and unprotected corridor railings. These environmental issues directly increase the risk of injuries among children. Additionally, the lack of traffic signals on roads near some communities has led to frequent traffic crashes. Timely identification and elimination of such hazardous environments are essential measures for preventing unintentional injuries among children.

“I hope the school can upgrade the playground. The current cement ground often grows moss, and many classmates have slipped.” [#120 Left-behind boy]

“The cafeteria floor often has water accumulation, and many students have slipped. I hope the teachers can add anti-slip mats.” [#112 Left-behind girl]

“The road near our community lacks traffic lights, leading to frequent accidents. I suggest installing traffic signals as soon as possible to ensure pedestrian safety.” [#75 Mother of a child with disabilities]

### 3.5.2 Increased resource support

Multiple respondents highlighted a significant gap between current resource allocation and actual needs. Communities and schools lack sufficient support in funding, staffing, materials, and professional training, resulting in limited service coverage and monotonous activity content. Adequate resource support would not only expand the scope of services but also improve the quality of activities, thereby more effectively preventing child injuries.

“Due to funding shortages, our social service agency can only serve about 100 of the 300 children in the community. To improve this, securing more financial support is crucial, only then can we develop higher-quality activities.” [#79 Social Worker]

“Teachers currently have too few opportunities for professional training on unintentional injuries among children. We hope to see more training in the future.” [#139 School Administrator]

### 3.5.3 Enhanced publicity efforts

The reach of safety education directly influences the safety awareness of children and parents. Currently, knowledge about preventing unintentional injuries is primarily disseminated through the education system, with limited involvement from media and social publicity channels. Participants suggested enhancing the systematic and engaging nature of publicity through lectures, emergency drills, case videos, and other formats to help children and parents gain a deeper understanding of safety knowledge.

“Awareness regarding the prevention of unintentional injuries remains insufficient in society, and there is an urgent need to strengthen publicity.” [#149 School Teacher]

“My knowledge of preventing child unintentional injuries is limited, I’ve only seen some fragmented videos on Douyin. I hope the school or community can organize relevant lectures.” [#70 Mother of a non-left-behind child]

“Children have short memory retention. It is necessary to repeatedly explain safety knowledge to them using real cases.” [#39 Father of a left-behind child]

### 3.5.4 Strengthened collaboration among all parties

Preventing and controlling unintentional injuries among children requires joint efforts from the government, communities, schools, and families. Participants mentioned that current collaboration is insufficient, and resource integration is weak, leading to limited effectiveness. There is an urgent need to establish a government-led cross-sector collaboration mechanism. “Child safety prevention and control should be led by the government, integrating efforts from communities, schools, and other stakeholders to form a joint prevention and control framework.” [#145 Social Worker] Additionally, home-school coordination needs strengthening: “There should be a focus on enhancing safety education for parents to improve their protective awareness and capabilities.” [#81 Social Worker]

“I suggest that schools collaborate with traffic police to arrange officers or volunteers to monitor the road in front of the school during student drop-off and pick-up times.” [#53 Mother of a child with disabilities]

### 3.5.5 Improved public facilities

Some public facilities in Guizhou Province, such as excessively steep staircases, increase the risk of falls for the older adults and children. For example, one participant noted: “Some underground passages in Guiyang have staircases that are too steep and high, making it inconvenient for the older adults and children to walk and easily causing falls. I hope future designs can be gentler.” [#74 Father of a non-left-behind child]

Participants also highlighted the challenges faced by children with disabilities. A teacher from a disability institution stated: “accessible facilities are nominal, with common issues such as occupied braille paths and overpasses lacking accessible ramps, threatening the daily safety of special needs children.” [#132 Teacher from a disability institution]

Another suggestion was: “Safety guarantees for children with disabilities should be strengthened. We hope relevant departments can improve the design and maintenance of accessible facilities to create an inclusive and safe environment.” [#91 Social Worker from a disability institution].

### 3.5.6 Innovation and upgrade of prevention methods

Participants reported that currently available safety education activities, such as standard lectures or posters, are relatively uniform in form and content, making it difficult to sustain the participation of children and parents. Participants called for technological innovation and service upgrades to design more engaging activity formats. Examples include developing smart warning systems to monitor hazardous areas in real time or introducing novel and interesting safety education activities to enhance participant engagement and publicity effectiveness.

“We are collaborating with a telecommunications company to develop a smart warning system that uses electronic school ID cards to monitor hazardous areas in real time.” [#123 School Administrator]

“Community safety promotions are basically the same as those at school. I’ve participated in them all and have lost interest. I hope there can be some new and fun activities.” [#115 Left-behind boy]

“Existing safety education activities lack innovation in both form and content, making it difficult to maintain children’s interest. We hope to design more novel and engaging activities to attract greater participation from children.” [#84 Social Worker]

## 4 Discussion

This study employed qualitative research methods to systematically explore the current status, effectiveness, gaps, and needs regarding interventions for the prevention and control of unintentional injuries among children aged 3–12 in Guizhou Province. Our findings indicate that the current prevention system has achieved certain successes, as reported by stakeholders. Crucially, these reports of effectiveness are not isolated; they resonate strongly with objective evidence from the global injury prevention literature. For instance, participants’ observations on the value of environmental modifications (e.g., installing corner guards, anti-slip flooring) are supported by systematic reviews confirming the efficacy of such safety devices in reducing the risk and severity of injuries (24). Similarly, the reported reduction in drowning incidents following the implementation of joint patrols aligns with studies identifying enhanced supervision and restricted access to hazardous water bodies as a cornerstone of drowning prevention (25, 26). Furthermore, the perceived improvement in children’s safety awareness through educational programs is consistent with evaluations demonstrating that school-based safety education can significantly increase children’s safety knowledge and improve safety practices (27). Therefore, while our data on effectiveness are self-reported, they are substantiated by existing empirical research.

However, our analysis reveals that the prevention system still faces numerous challenges, such as inadequate family supervision, prominent environmental hazards, insufficient publicity, resource shortages, and poor interdepartmental coordination. Among these, inadequate family supervision is a key factor affecting intervention effectiveness, which is consistent with previous studies (28, 29). The uniqueness of Guizhou lies in the dual constraints of economic pressure and household registration policies(commonly known as the hukou system, which restricts migrants’ access to social services, such as schooling, outside their registered home region) (30), which have created a distinct supervisory dilemma: a large number of children are forced into single-parent care, grandparent care, or even left to live alone due to parental labor migration (31, 32). This lack of supervision is not simply parental negligence but a choice under structural poverty. This phenomenon echoes the pattern of child injuries in rural India caused by seasonal migration (33), suggesting that developing countries face common structural risks.

Further analysis reveals a noticeable “technocratic tendency” in existing interventions, overreliance on material measures (e.g., installing protective facilities, providing electronic devices) while neglecting the deep influence of socio-cultural factors. For instance, traditional parenting beliefs, such as “it’s normal for children to get bumps and bruises,” widely held in rural areas, significantly hinder the adoption and effectiveness of safety measures. This finding aligns closely with Bourdieu’s (1990) theory of “habitus” (34), an unconscious attitude shaped by long-term living conditions (e.g., extensive parenting practices), which normalizes children’s “resilience to falls” and reduces willingness to adopt safety measures. This indicates that the formation of children’s safety behaviors is deeply influenced by specific socio-cultural contexts.

Regarding the needs for unintentional injury prevention and control interventions, Guizhou currently exhibits three distinct characteristics: First, needs are diverse, with different groups requiring tailored interventions. For example, left-behind children’s families urgently need economic support and specialized services; families of children with disabilities focus more on barrier-free environmental modifications and guardianship skills training; and local social organizations generally report shortages of professional talent and uneven resource distribution. Additionally, some participants expressed expectations for innovative prevention methods, such as electronic school card-based risk warning systems. Second, needs are precise, as traditional blanket interventions fail to meet the actual needs of specific groups. Third, needs are comprehensive, meaning that reliance on isolated efforts of a single department cannot fundamentally solve the problem, strengthened multi-sector collaboration is essential. These findings are highly consistent with the “targeted prevention” concept proposed by Winston et al. (35, 36).

The study further reveals a structural imbalance between current interventions and actual needs, mainly manifested in four aspects: First, there is a clear urban–rural gap in resource allocation, with significantly insufficient prevention and control resources in rural areas. Second, frontline social workers generally lack systematic training. Third, cross-sector collaboration mechanisms remain underdeveloped, often becoming mere formalities. Fourth, services for special groups, such as children with disabilities and left-behind children living alone, are inadequately covered. These issues are corroborated by findings from Sawant et al. (37, 38) in rural India, indicating that this is a common challenge faced by developing countries.

Based on the findings, this study recommends three key shifts in future prevention and control efforts: from extensive blanket coverage to targeted need satisfaction, from fragmented single-point breakthroughs to systematic holistic strategies, and from government-led approaches to multi-stakeholder governance. Specifically, it is necessary to strengthen family responsibility and improve the guardian support system; establish a tiered support system to meet the needs of special groups; increase resource investment to address environmental hazards and professional shortcomings; promote the development of smart warning systems to enhance risk prevention capabilities; set up special renovation funds to prioritize support for weak links; and improve collaboration mechanisms to build a government-led, multi-stakeholder prevention and control network. It is particularly emphasized that the effective implementation of these measures requires not only policy support but also a breakthrough from traditional thinking patterns to achieve organic integration of technical interventions and cultural guidance.

The findings of this study, while context-specific to Guizhou, offer insights that may be applicable to regions sharing similar socio-demographic and economic profiles. The identified challenges, such as the injury risks associated with left-behind children due to parental migration, the environmental hazards prevalent in rural and rapidly urbanizing areas, and the constraints faced by frontline social workers with limited resources, are not unique to Guizhou. Other provinces in China and settings in other low- and middle-income countries that experience large-scale labor migration, have significant rural-to-urban transitions, and operate with constrained public health resources are likely to face analogous challenges. Therefore, the themes and intervention needs identified in this “bottom-up” inquiry could serve as a valuable framework for understanding contexts and designing preliminary strategies in those comparable settings.

This study has several limitations. First, as a qualitative study, its conclusions are primarily based on participants’ subjective statements and researchers’ interpretive analysis, which may be susceptible to social desirability bias or researcher influence, despite our efforts to mitigate these through rigorous coding procedures. Second, we explicitly acknowledge that a mixed-methods design, incorporating structured quantitative surveys, could have provided complementary objective data. However, the primary aim of this research was foundational and exploratory: to map the uncharted landscape of challenges and needs from the “bottom-up” perspectives of stakeholders themselves. Our findings now provide the essential conceptual framework to guide the future development of meaningful quantitative instruments. Third, the sample coverage was relatively limited, insufficiently including children from remote mountainous areas, and no quantitative evaluation of the effectiveness of current prevention and control measures was conducted. Despite these limitations, this study offers invaluable in-depth insights that establish a critical evidence base for understanding the status and needs of child injury prevention in Guizhou. Subsequent studies are recommended to adopt mixed-methods approaches, expand sample coverage, and incorporate longitudinal designs to evaluate intervention effects, thereby providing stronger evidence for establishing a more robust child safety protection system.

## 5 Conclusion

The prevention and control of unintentional injuries among children aged 3–12 in Guizhou Province presently comprises an intervention system primarily based on safety education, joint prevention and control, and physical protection under government leadership, which has to some extent enhanced children’s safety awareness and reduced injury occurrences. However, the current efforts still face multiple structural challenges, including inadequate family supervision, prominent environmental and facility hazards, insufficient resource allocation, and poor interdepartmental coordination. Vulnerable groups such as left-behind children living alone and children with disabilities face higher risks of injury. Based on diversified, precise, and systematic prevention and control needs, future efforts should transition from extensive coverage to targeted interventions. By strengthening family supervision functions, resource support, technological empowerment, and multi-party collaboration, a prevention and control system aligned with the cultural characteristics of Guizhou’s multi-ethnic regions should be constructed.

## Data Availability

The qualitative data from this study is available to bona fide researchers upon request from the corresponding author. Access to the data is controlled to protect the privacy of participants and the community.
