# Supplementary material for: A qualitative study on the current status and needs of unintentional injury prevention and control interventions for children aged 3–12 in Guizhou Province, China
Source: Front Public Health. 2025 Dec 8;13:1712091. doi: 10.3389/fpubh.2025.1712091 (PMC12722910; doi:10.3389/fpubh.2025.1712091)
Supplement: Supplementary file 1 [file Table_1.docx]

****Interview Outline****

****Title: A Qualitative Study on the Current Status and Needs of Unintentional Injury Prevention and Control Interventions for Children Aged 3-12 in Guizhou Province, China****

****I. Focus Group Discussions****
****Participants: Children aged 3–12 (children capable of clearly expressing their thoughts, 5–6 per group)****

****1. Children’s Understanding and Current Status of Unintentional Injury Prevention and Control****
(1) Have you been injured in the past year (12 months)? Where did it happen? Why did it happen? How did you handle the injury after it occurred?
(2) What types of unintentional injuries are you aware of?
(3) What methods do you usually use to protect yourself from getting injured (e.g., how to avoid burns or falls)? How did you learn these methods?
(4) What measures do you know that protect children’s safety? Where have you seen these measures (at home, school, or in the community)? What do you think these measures do?
(5) Has anyone taught you about safety? Who teaches you? How do they teach you (e.g., videos, pictures, role-playing, or verbal instruction)? Can you give examples? Anything else?
(6) What activities have you participated in related to preventing unintentional injuries among children? Where did you participate (school or community)? How were these activities conducted (e.g., videos, lectures, verbal reminders, drills)?

****2. Evaluation of the Effectiveness of Existing Interventions****
(7) Do you think these safety promotion activities are useful? Why?
(8) Which intervention measures do you think are more effective? Why?

****3. Shortcomings in Prevention and Control and Related Needs****
(9) Which areas at home do you think are dangerous and likely to cause injuries? Why? (e.g., slippery floors, sharp furniture edges, other reasons)
(10) In terms of protecting your safety, what do you think your parents could do better? Why? How would you like them to improve?
(11) Which areas in school do you think are dangerous and likely to cause injuries to students? Why?
(12) In terms of protecting students’ safety, what do you think teachers could do better? Why? How would you like them to improve?
(13) Which areas in the community do you think are dangerous and likely to cause injuries? Why?
(14) In terms of protecting people’s safety, what do you think community workers could do better? Why? How would you like them to improve?

****4. Closing****
(15) Is there anything else you would like to add?

****II. In-depth Interviews****
****Participants: Child Caregivers****

****1. Caregivers’ Understanding and Current Status of Unintentional Injury Prevention and Control****
(1) Has your child been injured in the past year (12 months)? What type of injury was it? Where did it happen? Why did it happen?
(2) What types of unintentional injuries in children are you aware of?
(3) What safety knowledge do you teach your child? How do you usually educate them (e.g., watching videos, verbal reminders)?
(4) Which areas at home do you think are dangerous and likely to cause injuries to children? Why? (e.g., slippery floors, sharp furniture edges, other reasons)
(5) Have you taken any measures to prevent unintentional injuries in children? What specific measures have you taken? Please provide examples (e.g., covering sharp table edges, installing socket covers).
(6) Has your child been injured at school or in the community? What was the cause? How did you find out about the injury? What did you do after finding out?
(7) What efforts have the school and community made in preventing unintentional injuries in children? How were these efforts carried out? How effective do you think they are? What areas need improvement, and how?

****2. Evaluation of the Effectiveness of Existing Interventions****
(8) How effective are the current intervention measures? Can you provide examples?
(9) In your opinion, which intervention measures are more effective? Are there specific cases?

****3. Shortcomings in Prevention and Control****
(10) In what areas could your family improve in ensuring child safety? How would you like to see improvements?
(11) Do you think your safety knowledge is sufficient? If needed, what safety knowledge or skills would you like to learn more about?
(12) In your opinion, how does society view and pay attention to unintentional injuries in children? Why? Can you give an example?

****4. Needs in Prevention and Control****
(13) What are your urgent needs regarding the prevention and control of unintentional injuries in children? Why?
(14) In your opinion, which aspects of unintentional injury prevention and control need the most strengthening? Why?
(15) Based on the actual situation in Guizhou, what specific suggestions do you have for preventing and controlling unintentional injuries in children?

****5. Multi-Stakeholder Collaboration****
(16) How do you think the community, school, government, and families should collaborate in prevention and control efforts?
(17) What current forms or channels of collaboration exist between families and social organizations, schools, and the government? Do you think it is necessary to strengthen collaboration further? Why? How should collaboration be enhanced?

****6. Closing****
(18) Is there anything else you would like to add regarding the prevention and control of unintentional injuries in children?

****III. In-depth Interviews****
****Participants: Teachers of Children Aged 3–12 (Including School Administrators)****

****1. School Administrators’ Understanding and Current Status of Unintentional Injury Prevention and Control****
(1) What types of unintentional injuries in children are you aware of?
(2) What specific measures has the school taken to prevent unintentional injuries in children?
(3) How are these measures implemented? What is the frequency of implementation? What is the coverage of these services?

****2. Evaluation of the Effectiveness of Existing Interventions****
(4) How effective are the current intervention measures? Are there any data or cases to support this?
(5) In your opinion, which intervention measures are more effective? Are there specific cases or data to support this?

****3. Shortcomings in Prevention and Control****
(6) What shortcomings do you see in the school’s current intervention measures? How do these shortcomings affect prevention and control efforts? How do you suggest optimizing them?
(7) Do teachers have sufficient professional knowledge in preventing and controlling unintentional injuries in children? Which competencies need urgent improvement?

****4. Needs in Prevention and Control****
(8) What are the school’s urgent needs in preventing and controlling unintentional injuries in children?
(9) In your opinion, which aspects of unintentional injury prevention and control need the most strengthening?
(10) Based on the actual situation in Guizhou, what specific suggestions do you have for preventing and controlling unintentional injuries in children?

****5. Multi-Stakeholder Collaboration****
(11) How do you think the community, school, government, and families should collaborate in prevention and control efforts?
(12) What current forms or channels of collaboration exist between the school and social organizations, the government, and families? Do you think it is necessary to strengthen collaboration further? Why? How should collaboration be enhanced?

****6. Closing****
(13) Is there anything else you would like to add regarding the prevention and control of unintentional injuries in children?

****IV. In-depth Interviews****
****Participants: Social Workers (Including Social Work Managers)****

****1. Social Workers’ Understanding and Current Status of Unintentional Injury Prevention and Control****
(1) What types of unintentional injuries in children are you aware of?
(2) Has your social work agency taken any measures to prevent unintentional injuries in children? What specific measures have been taken?
(3) How are these measures implemented in the community? What is the frequency of implementation? What is the coverage of these services?

****2. Evaluation of the Effectiveness of Existing Interventions****
(4) How effective are the current intervention measures? Are there any data or cases to support this?
(5) In your opinion, which intervention measures are more effective? Are there specific cases or data to support this?
(6) What unique role do you think social work agencies play in prevention and control efforts?

****3. Shortcomings in Prevention and Control****
(7) What shortcomings do you see in the current intervention measures of social work agencies? How do these shortcomings affect prevention and control efforts? How do you suggest optimizing them?
(8) Do social workers have sufficient professional knowledge in preventing and controlling unintentional injuries in children? Which competencies need urgent improvement?
(9) In your opinion, how does society view and pay attention to unintentional injuries in children? Why? Can you give an example?

****4. Needs in Prevention and Control****
(10) What are the community’s urgent needs in preventing and controlling unintentional injuries in children?
(11) In your opinion, which aspects of unintentional injury prevention and control need the most strengthening?
(12) Based on the actual situation in Guizhou, what specific suggestions do you have for preventing and controlling unintentional injuries in children?

****5. Multi-Stakeholder Collaboration****
(13) How do you think the community, school, government, and families should collaborate in prevention and control efforts?
(14) What current forms or channels of collaboration exist between social organizations and schools, the government, and families?
(15) How can collaboration among these stakeholders in prevention and control efforts be further strengthened?

****6. Closing****
(16) Is there anything else you would like to add regarding the prevention and control of unintentional injuries in children?
